# Dosimetric comparison of intensity‐modulated proton therapy and photon‐based radiotherapy for skull base tumors

**DOI:** 10.1002/acm2.70268

**Published:** 2025-10-13

**Authors:** Yuanyuan Wang, Jiajun Zheng, Lei Liu, Yuxiang Wang, Tengfei Long, Ming Liu, Jin Gao, Hsiao‐ming Lu

**Affiliations:** ^1^ Hefei Ion Medical Center, the First Affıliated Hospital of USTC, Division of Life Sciences and Medicine University of Science and Technology of China Hefei China; ^2^ Department of Radiation Oncology the Affiliated Cancer Hospital of Nanjing Medical University Nanjing China; ^3^ Institute of Health and Medical Technology University of Science and Technology of China Hefei China; ^4^ Hefei Institutes of Physical Science Chinese Academy of Sciences Hefei China; ^5^ Department of Radiation Oncology, the First Affiliated Hospital of USTC, Division of Life Sciences and Medicine University of Science and Technology of China Hefei China

**Keywords:** dosimetry, IMPT, proton therapy, skull‐base tumor

## Abstract

**Objective:**

To compare dosimetric differences between intensity‐modulated proton therapy (IMPT) and photon‐based radiotherapy (VMAT/IMRT) in the treatment of skull base tumors, focusing on target coverage and organ‐at‐risk (OAR) sparing.

**Methods:**

All patients with skull base tumors who underwent proton therapy between December 2021 and August 2022 were included in this study. For each case, both IMPT and VMAT/IMRT plans were generated using Eclipse TPS, with robust optimization applied for protons. Dosimetric metrics for target volumes and OARs—including the brainstem, spinal cord, visual pathways, eyes, lenses, and salivary glands—were extracted and compared. Key endpoints included $D_{2\%}$, $D_{\text{mean}}$, conformity index (CI), and homogeneity index (HI). Statistical comparisons were performed using non‐parametric Wilcoxon signed‐rank tests, with significance set at *p*<0.05.

**Results:**

Target coverage was comparable between IMPT and photon plans ($D_{98\%}$ of 57.51±6.14 Gy for photon and 58.06±5.85 Gy for proton, *p* = 0.12, respectively). IMPT provided statistically significant dose reductions to brainstem, spinal cord, lenses, eyes, and the right parotid. The brainstem $D_{2\%}$ was reduced from 45.70±11.49 Gy of photons to 39.47±13.03 Gy of protons (*p* = 0.037). Dose reductions of ∼29%–83% were observed for other above OARs. Although no significant difference was found in doses to the left parotid or visual pathways, substantial dose reductions were observed in individual cases.

**Conclusion:**

IMPT achieved equivalent target coverage to VMAT/IMRT while offering superior sparing of several critical OARs. These findings support the clinical potential of IMPT in the management of anatomically complex skull base tumors, particularly when normal tissue preservation is a key concern.

## 1 INTRODUCTION

Skull base tumors encompass both primary neoplasms (such as chordomas, meningiomas, and pituitary adenomas)^1^, ^2^, ^3^ and the local invasion by head and neck tumors.^4^, ^5^ Radiation therapy serves as a cornerstone therapeutic modality, either as definitive treatment or in conjunction with surgical resection.^6^ However, the skull base represents an anatomically intricate and functionally vital region, housing critical neurovascular structures, including the brainstem, spinal cord, major cerebral vasculature, and multiple cranial nerves (such as the visual pathway)^7^, rendering meticulous management of radiation‐induced toxicities particularly crucial in therapeutic planning and delivery.^8^, ^9^ Recent advances in techniques such as MRI‐guided adaptive radiotherapy^10^, proton therapy, and carbon particle therapy^11^ have significantly enhanced treatment precision while reducing risks to adjacent critical structures. Furthermore, integrating molecular biomarkers and radiomics for personalized dose optimization remains underexplored, underscoring critical gaps in balancing oncological efficacy with functional preservation.^12^

Proton therapy has gained traction in skull base oncology due to its unique physical properties—the Bragg peak energy deposition and sharp dose fall off—which theoretically maximize tumor targeting while sparing adjacent organs‐at‐risk (OARs). Clinical studies have reported a promising 5‐year progression‐free survival (PFS) rate of 74% (range: 45%–100%, *n* = 102) for skull‐base chordomas treated with proton therapy, which is substantially higher than the 35% (range: 26%–45%, *n* = 95) observed with photon therapy, reflecting improved conformity and disease control.^13^ Patients with meningiomas and pituitary adenomas often achieve excellent long‐term tumor control after radiotherapy, with reported 10‐year local control rates exceeding 80%.^14^, ^15^ Given this favorable prognosis, the focus of treatment extends beyond disease eradication to minimizing long‐term toxicities that may impair neurocognitive, visual, auditory, or endocrine functions. As such, modern treatment planning should emphasize not only conformal tumor coverage but also the preservation of quality of life through optimal sparing of adjacent critical neural structures.

Despite encouraging clinical outcomes, the current evidence base for proton therapy in skull base tumors remains limited and heterogeneous. Most published studies are retrospective, single‐institution series with small sample sizes and inconsistent follow‐up durations (typically 3–5 years). These limitations highlight the urgent need for systematic, quantitative evaluations of proton therapy's dosimetric benefits in complex anatomical settings such as the skull base. In light of these uncertainties, dosimetric studies remain a valuable tool to objectively quantify the physical advantages of proton therapy in complex anatomical settings. Skull base tumors present a particularly relevant context for such comparison, given the proximity of target volumes to radiosensitive structures such as the brainstem, optic apparatus, and cranial nerves. To date, few reports have directly contrasted intensity‐modulated proton therapy (IMPT) with advanced photon techniques—such as intensity‐modulated radiation therapy (IMRT) or volumetric modulated arc therapy (VMAT)—in a uniform cohort of skull base cases.

This study aims to perform a detailed dosimetric comparison between IMPT and VMAT/IMRT plans in patients with skull base tumors, using standardized target coverage criteria and organ‐at‐risk (OAR) constraints. Our findings may inform individualized modality selection and support future trial designs grounded in dose‐based rationale.

## 2 METHODS

### 2.1 Patient cohort

All 12 patients (seven male and five female) with skull base tumors who underwent proton therapy at Hefei Ion Medical Center (HIMC) between December 2021 and August 2022 were included in this study. Table 1 shows the clinical, demographic, and prescription information for the study cohort. The median age at diagnosis was 40.5 years (range: 30–69 years). Specifically, there were five chordomas (located in the clivus or upper cervical spine), two meningiomas, one pituitary adenoma, three adenoid cystic carcinomas (ACC) involving the skull base, and one malignant nasal cavity tumor with skull base extension. In all cases, the target volumes were anatomically complex and in close proximity to critical OARs, underscoring the need for precise conformal dose delivery. This study was approved by the institutional review board, and individual patient consent was waived due to the retrospective nature and use of de‐identified data.

**TABLE 1:** Clinical, demographic, and prescription information of the study cohort.

Case	Sex	Age	Site	Status	GTV	CTV
#1	female	30	Chordomas	Primary	70 Gy (35f)	64 Gy (32f)
#2	male	60	Chordomas	Primary	70 Gy (35f)	56 Gy (28f)
#3	female	30	ACC	Primary		70 Gy (35f)
#4	female	35	Pituitary adenoma	Recurrent		50.4 Gy (28f)
#5	male	67	Meningiomas	Recurrent		54 Gy (30f)
#6	female	60	Meningiomas	Primary		54 Gy (27f)
#7	female	39	Nasal cavity	Primary	60 Gy (28f)	56 Gy (28f)
#8	male	39	Chordomas	Recurrent	70 Gy (35f)	56 Gy (28f)
#9	male	59	Chordomas	Recurrent	70 Gy (35f)	56 Gy (28f)
#10	male	39	Chordomas	Primary	70 Gy (35f)	56 Gy (28f)
#11	male	42	ACC	Primary		56 Gy (28f)
#12	male	69	ACC	Primary	66.03 Gy (31f)	59.52 Gy (31f)

Abbreviations: ACC, adenoid cystic carcinomas; CTV, clinical target volume; GTV, gross tumor volume.

For each patient, target volumes were defined according to standard practice. Planning CT scans (Siemens Healthcare, Germany) were acquired with slice thicknesses of 2 mm for all 12 patients. The gross tumor volume (GTV) and clinical target volume (CTV) were delineated by experienced radiation oncologists based on fusion of the CT and MRI. Dose prescription was individualized based on treatment intent, tumor dimensions, and specific clinical considerations for each patient. For the chordoma patients, an integrated boost approach was used with a higher dose (70 Gy in 35 fractions) to the GTV and a lower dose (56 Gy/64.0 Gy in 28/32 fractions) to the surrounding CTV. The benign tumors (meningiomas and pituitary adenomas) were treated with a single prescription level covering the entire target (54 Gy in 27–30 fractions for meningioma, 50.4 Gy in 28 fractions for pituitary adenoma). For patients with ACC, dose prescriptions varied according to individual clinical indications and tumor extent. Several fractionation schemes were employed, including 56–70 Gy in 28–35 fractions, as detailed in Table 1. A simultaneous integrated boost (SIB) prescription was used for the nasal cavity tumor with 60 Gy to GTV and 56 Gy to CTV in 28 fractions.

### 2.2 IMPT plans

Proton therapy plans were created using the pencil beam scanning (PBS) technique on the ProBeam facility in the Varian Eclipse treatment planning system (version 16.01). Beam arrangements typically consisted of two to three coplanar or noncoplanar fields, selected based on tumor location and proximity to critical structures. Spot spacing was automatically determined by Eclipse, typically ranging from 3.0 to 4.0 mm. Robust optimization was applied directly to the CTV, incorporating setup uncertainty of ±3 mm and range uncertainty of ±3%. A total of 12 perturbed scenarios were generated and optimized simultaneously to ensure plan robustness against anatomical and delivery variations. Worst‐case dose‐volume histogram (DVH) analysis was conducted for both targets and OARs to evaluate robustness post‐optimization. The number of energy layers and spot weights were optimized layer‐by‐layer to achieve conformal target coverage. All IMPT plans were calculated with the proton convolution–superposition (PCS) dose engine embedded in Eclipse v16.01. PCS is an analytical pencil‐beam convolution model: energy‐dependent dose kernels—incorporating both energy deposition and multiple‐Coulomb‐scattering broadening—are pre‐computed in water, then scaled to each voxel's water‐equivalent path length and convolved over a three‐dimensional grid to obtain the dose distribution. All doses were expressed in Gy(RBE), assuming a relative biological effectiveness (RBE) of 1.1. Plans were normalized such that at least 95% of the CTV received 100% of the prescribed dose under the nominal scenario.

### 2.3 IMRT/VMAT plans

The same target and OAR contours, prescription doses, and dose‐volume constraints to IMPT plans were used in photon plans to facilitate objective dosimetric comparison. IMRT/VMAT plans were created using the Varian Eclipse treatment planning system (version 15.6) on a TrueBeam linear accelerator platform equipped with a high‐definition multileaf collimator (HD120 MLC). Planning target volumes (PTVs) were generated by isotropically expanding the CTV by 3 mm. All plans employed used 6 MV photon beams. Beam arrangements typically consisted of multiple IMRT fields or two VMAT (full or partial) arcs, with gantry angles selected to optimize dose conformity and minimize exposure to adjacent organs‐at‐risk (OARs). Non‐coplanar arcs were not used. Inverse planning was conducted using the Photon Optimizer (PO), with optimization objectives defined for both targets and OARs. Optimization priorities were manually adjusted per patient to ensure adequate target coverage while respecting institutional dose constraints for normal tissues. Final dose calculation was performed using the anisotropic analytical algorithm (AAA) with a grid size of 2.5 mm. All plans were normalized such that at least 95% of the PTV received 100% of the prescription dose.

### 2.4 Dosimetry

Dose‐volume histograms (DVHs) were calculated for all targets and OARs. The dosimetric evaluation incorporated two primary categories of metrics: target coverage and OAR sparing. Target coverage assessment included $D_{98\%}$, $D_{\text{mean}}$, and $D_{2\%}$ for both GTV and CTV. The conformity index (CI) and homogeneity index (HI) were also calculated for each plan. CI was defined as the ratio of the volume of the prescription iso‐dose that overlaps the target to the volume of the prescription iso‐dose. HI was defined as $HI=D_{2\%}/D_{98\%}$. For OARs, the following structures were contoured and analyzed: (1) $D_{2\%}$ for brainstem, spinal cord, optic chiasm, optic nerves, and lenses; (2) $D_{\text{mean}}$ for eyes and parotid glands.

### 2.5 Statistical analysis

Each pair of plans (photon vs. proton for the same patient) was compared for the above metrics. To summarize across patients, descriptive statistics (mean ± standard deviation) were used. A paired statistical comparison was performed for each dosimetric endpoint to assess the significance of differences. Given the small sample size (*n* = 12) and non‐normal distribution of some parameters, we used the non‐parametric Wilcoxon signed‐rank test for most comparisons. A two‐tailed p<0.05 was considered statistically significant.

## 3 RESULTS

### 3.1 Dosimetry

**FIGURE 1:**
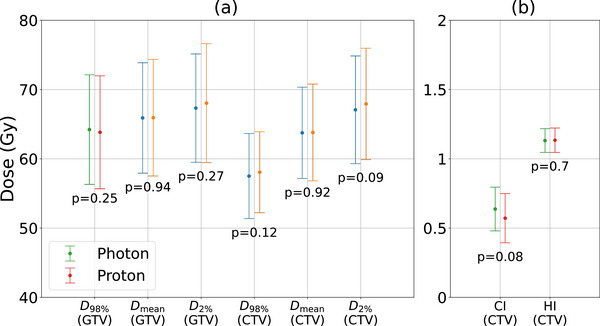
Comparison of target dose metrics and conformity/homogeneity indices between IMRT/VMAT and IMPT plans. IMPT, intensity‐modulated proton therapy; IMRT, intensity‐modulated radiation therapy; VMAT, volumetric modulated arc therapy.

Figure 1 gives comparison of target dose metrics and conformity/homogeneity indices between VMAT and IMPT. Both proton and photon plans achieved comparable target coverage ($D_{98\%}$ of 57.51±6.14 for photon and 58.06±5.85 for proton) with a difference that was not statistically significant (*p* = 0.12). The prescribed dose coverage of the CTV was not compromised in the proton plans, as is shown in Figure 1. CI of the CTV was 0.64±0.16 for photon plans and 0.57±0.18 for protons (*p* = 0.08). The CI value being less than 1 was attributed to the use of PTV margins or robustness optimization, which resulted in a larger prescription isodose volume than the actual CTV coverage required. These findings suggest that IMPT achieved comparable target coverage and conformity to that of IMRT/VMAT, fulfilling the primary objective of delivering the prescribed dose effectively to the tumor volume. The mean HI for the CTV was 1.13 ± 0.09 for photon and 1.13 ± 0.10 for proton (*p* = 0.7), indicating that both techniques produced a small degree of dose heterogeneity within the target. These differences balanced out and were deemed clinically insignificant.

Although target coverage was comparable between photon and proton plans, notable differences were observed in the dose delivered to OARs, as summarized in Table 2. Compared to photon therapy, proton plans provided statistically significant dose reductions to several critical structures, most prominently the brainstem, spinal cord, lenses, eyes, and right parotid gland. The magnitude of reduction ranged from 13.63% to 83.93%, depending on the OAR and anatomical proximity to the target. For instance, the mean $D_{2\%}$ of the brainstem was 39.47±13.03 Gy in IMPT plans, compared to 45.70±11.49 Gy in IMRT/VMAT (*p *= 0.011), corresponding to a 13.63% average reduction. Proton plans yielded lower maximum brainstem doses in 11 out of 12 patients, with a maximum reduction of 17.99 Gy observed in case #11. For the spinal cord, the mean $D_{2\%}$ decreased by over 70% with IMPT (3.84 vs. 14.49 Gy, *p *= 0.037), indicating significantly improved sparing. Substantial reductions were also seen for the lenses (up to 84%), eyes (mean reduction >60%), and right parotid (up to 63%), with all these differences reaching statistical significance. These findings emphasize the capability of IMPT to reduce unnecessary dose to adjacent normal tissues while maintaining equivalent target coverage.

**TABLE 2:** Quantitative dosimetric comparison of OARs between IMRT/VMAT and IMPT plans.

OAR	Metric	Photon	Proton	*p*‐Value	δ
Brainstem	$D_{2\%}$	45.70 ± 11.49	39.47 ± 13.03	0.011	−13.63%
Spinal cord	$D_{2\%}$	14.49 ± 15.59	3.84 ± 4.96	0.037	−73.49%
Len‐L	$D_{2\%}$	5.32 ± 2.15	0.86 ± 1.90	0.000	−83.93%
Len‐R	$D_{2\%}$	6.24 ± 3.84	3.12 ± 4.37	0.000	−49.95%
Eye‐L	$D_{\text{mean}}$	7.53 ± 4.91	2.34 ± 4.82	0.007	−68.91%
Eye‐R	$D_{\text{mean}}$	10.77 ± 10.03	7.56 ± 9.55	0.007	−29.82%
Parotid‐L	$D_{\text{mean}}$	6.65 ± 12.33	1.23 ± 2.55	0.102	✗
Parotid‐R	$D_{\text{mean}}$	9.44 ± 11.72	3.52 ± 7.35	0.040	−62.75%
Chiasm	$D_{2\%}$	33.66 ± 22.96	30.36 ± 20.66	0.172	✗
Opt‐L	$D_{2\%}$	33.65 ± 16.30	25.68 ± 19.18	0.063	✗
Opt‐R	$D_{2\%}$	40.70 ± 19.44	38.33 ± 18.68	0.26	✗

*Note*: δ = Diff (value of proton to value of photon)/value of photon.

Abbreviations: IMPT, intensity‐modulated proton therapy; IMRT, intensity‐modulated radiation therapy; OARs, organs‐at‐risk; VMAT, volumetric modulated arc therapy.

**FIGURE 2:**
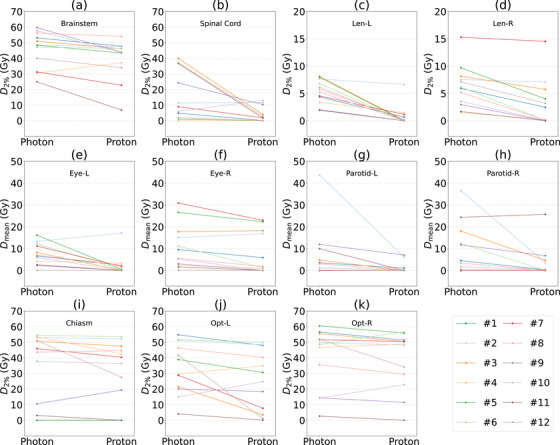
Dose comparison of OARs between IMRT/VMAT and IMPT plans. IMPT, intensity‐modulated proton therapy; IMRT, intensity‐modulated radiation therapy; OARs, organs‐at‐risk; VMAT, volumetric modulated arc therapy.

Although proton therapy achieved substantial dose reductions for several OARs, no statistically significant improvements were observed for the left parotid gland, the optic chiasm, or the bilateral optic nerves (Table 2). Figure 2 provides a patient‐level visualization of dosimetric differences between photon and proton plans for OARs especially the selected salivary (g) and visual structures (i–k). The relative dose sparing varied widely across patients, largely depending on tumor location and its spatial proximity to these structures. In certain cases, such as #12, proton plans achieved marked dose reductions to the above visual pathway, with dose reductions of ∼17–40 Gy. Notably, in case #2 in Figure 2g, the $D_{\text{mean}}$ decreased dramatically from 43.7 Gy with photon plan to 6.1 Gy with proton plan—a reduction of over 85%. Although these values remain within acceptable limits for a single course of radiotherapy, such reductions may prove clinically relevant in re‐irradiation settings or in patients with reduced organ tolerance. Collectively, these findings underscore that IMPT's advantages are highly dependent on tumor—OAR geometry, and may not translate to uniform benefit across all adjacent structures.

**FIGURE 3:**
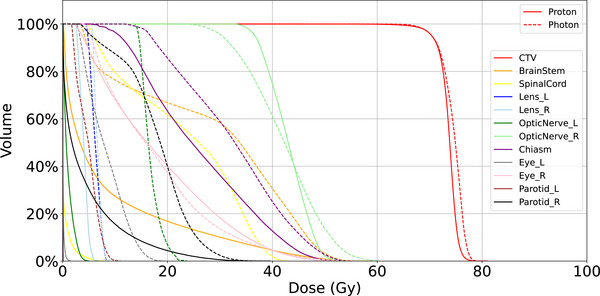
Representative dose‐volume histogram comparison for IMRT/VMAT and IMPT plans in Case #3. IMPT, intensity‐modulated proton therapy; IMRT, intensity‐modulated radiation therapy; VMAT, volumetric modulated arc therapy.

**FIGURE 4:**
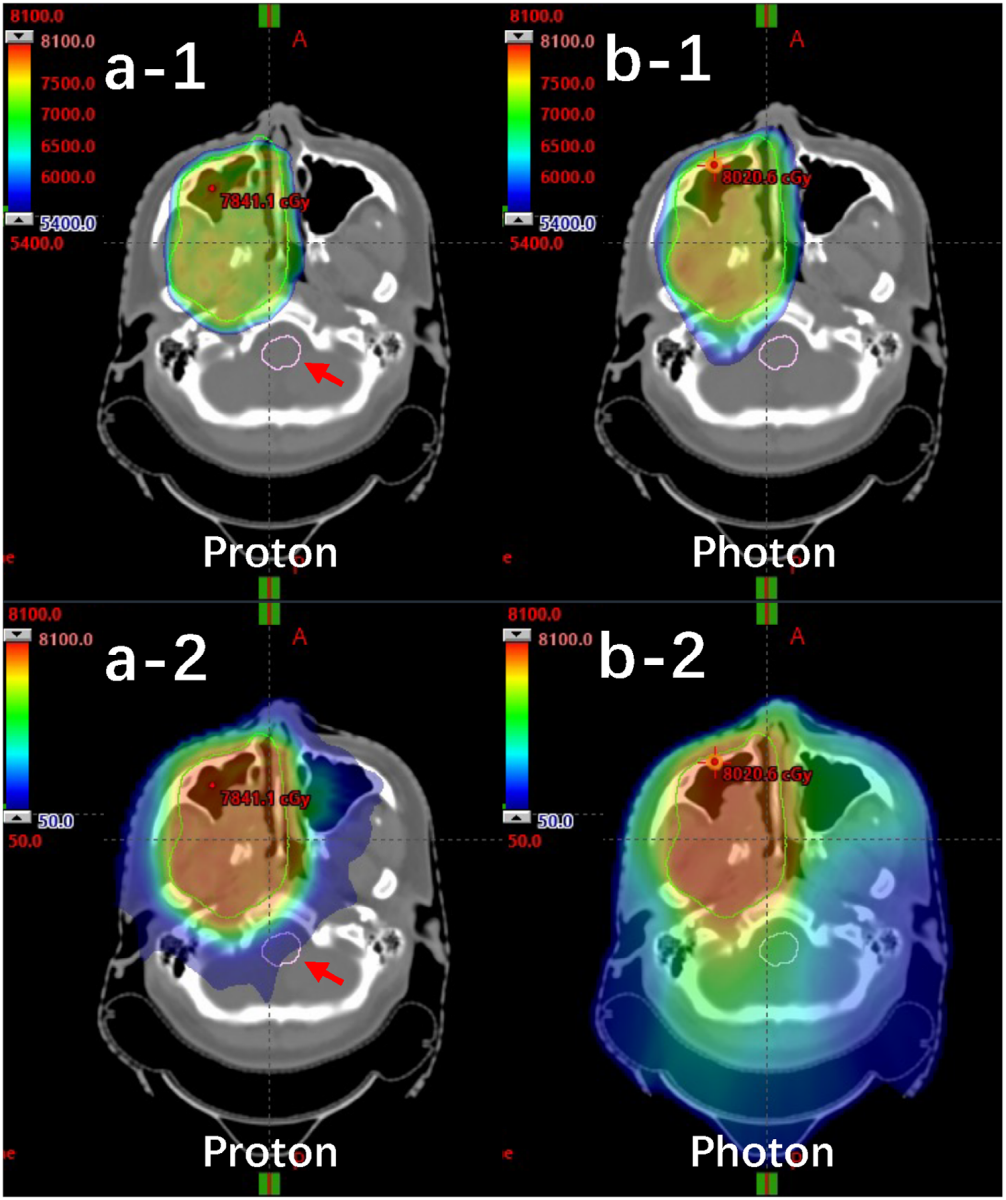
Representative isodose distributions for IMPT (a‐1 and a‐2) and IMRT/VMAT (b‐1 and b‐2) plans in case #3. IMPT, intensity‐modulated proton therapy; IMRT, intensity‐modulated radiation therapy; VMAT, volumetric modulated arc therapy.

Figure 3 presents a comparative dose‐volume histogram (DVH) analysis for case #3, illustrating dose metrics for CTV and multiple OARs under photon (IMRT/VMAT) and proton (IMPT) plans. As shown, both modalities achieved comparable CTV coverage, with nearly overlapping DVH curves, affirming that the improved normal tissue sparing of IMPT did not come at the cost of target underdosage. However, marked differences in OAR sparing were evident across several structures. The DVHs demonstrate consistently lower doses to the brainstem, spinal cord, optic nerves, lenses, and parotid glands with protons. This dosimetric advantage is further exemplified in the isodose distributions shown in Figure 4. In panels (a‐1) and (a‐2) (proton plan), the sharp dose fall‐off and conformality of IMPT can be appreciated, particularly around the brainstem and optic structures. In contrast, panels (b‐1) and (b‐2), representing the photon plan, show more diffuse low‐to‐intermediate dose spread due to the nature of multi‐beam IMRT/VMAT delivery. Case #3 thus serves as a clear example of how IMPT can achieve similar tumor control objectives with superior OAR protection in anatomically challenging settings.

## 4 DISCUSSION

This study presents a comparative dosimetric analysis of IMPT versus photon therapy (IMRT/VMAT) for skull base tumors in a cohort of 12 patients. Our results are consistent with the inherent physical properties of protons, notably the Bragg peak and absence of exit dose, enabling steep dose gradients that reduce irradiation to surrounding normal tissues. By incorporating comprehensive OAR dosimetric metrics, our study offers critical insight into how IMPT can enhance the therapeutic ratio in anatomically complex and high‐risk skull base tumor scenarios.

This dosimetric comparison of IMPT and advanced photon radiotherapy (VMAT/IMRT) for skull base tumors demonstrates that both modalities can achieve equivalent target coverage, with comparable conformity and homogeneity. However, IMPT significantly reduces dose to critical OARs, including the brainstem, spinal cord, eyes, lenses, and parotid glands. These advantages stem from the physical characteristics of protons—particularly the Bragg peak and minimal exit dose—which enable steep dose gradients and less irradiation to surrounding tissues. The observed brainstem dose reductions are particularly relevant, given the proximity of many skull base tumors to this critical structure. Even modest decreases in brainstem dose may reduce the risk of necrosis or cranial neuropathy.^16^ Similarly, reductions in lens and ocular doses may mitigate long‐term risks of radiation‐induced cataracts or retinopathy^17^, ^18^, while lower parotid doses may help preserve salivary function and reduce xerostomia.^19^ Our findings are consistent with retrospective series and planning studies showing that proton therapy can deliver high tumoricidal doses with acceptable toxicity, especially for chordomas and chondrosarcomas.^20^, ^21^, ^22^ Although large‐scale randomized trials are lacking, the consistent dosimetric advantages of IMPT strongly suggest potential clinical benefit, including reduced late effects and improved quality of life. However, further prospective studies are needed to confirm whether these dosimetric improvements translate into better patient outcomes. Taken together, our results support the growing consensus that IMPT is a preferred modality for skull base tumors, particularly when adjacent to dose‐sensitive structures.

Multiple planning studies have shown that IMPT can significantly reduce radiation exposure to critical OARs—such as the brainstem, optic apparatus, and contralateral parotid gland—without compromising target coverage.^23^ Hirano et al. demonstrated improved sparing of the brainstem and optic pathways with proton therapy^24^, while other reports noted substantial reductions and near‐zero dose to the contralateral parotid.^25^, ^26^, ^27^ In our cohort, similar patterns were observed, with proton plans achieving markedly lower doses to both ipsilateral and contralateral salivary glands. These findings have important clinical implications, particularly in the context of reducing the incidence and severity of radiation‐induced xerostomia, thereby improving long‐term outcomes in speech, nutrition, and oral health. More broadly, the ability of protons to reduce integral dose may help lower the risk of late toxicities such as soft tissue fibrosis, ototoxicity, pituitary dysfunction, and potentially secondary malignancies. To validate whether these dosimetric advantages translate into tangible clinical benefits, future studies should incorporate prospective, longitudinal follow‐up assessing late toxicity, neurocognitive function, and patient‐reported quality of life outcomes in patients receiving IMPT versus photon‐based radiotherapy.

Although IMPT showed clear dosimetric advantages for several OARs, no statistically significant reductions were observed for the optic pathways (the optic chiasm and optic nerves) compared to VMAT. This reflects a key clinical consideration: when critical structures abut or overlap the target, both modalities are constrained by similar tolerance limits. Among the 12 patients, the left optic nerve was either encompassed by the target volume or located within 5 mm (this distance referred to the minimum of the closest distances between the target volume and the OARs on each slice, manually measured by an experienced medical physicist) of it in seven cases. The right optic nerve was similarly involved in 10 cases. As for the optic chiasm, it was encompassed or located within 5 mm of the target in 9 cases as well. Notably, only in case #11 was the target located more than 2 cm away from all three visual pathway structures. In such cases, the theoretical benefit of proton therapy—especially its lack of exit dose—may be offset by range uncertainty margins and robustness requirements.^28^ Some studies have even shown that IMRT, using multiple beam angles, can achieve lower point doses to certain adjacent OARs.^29^ These findings underscore that IMPT is not inherently superior in all situations; its advantage depends on patient‐specific anatomy and planning feasibility. Therefore, treatment selection should be based on individualized plan comparisons and, where possible, informed by model‐based frameworks incorporating NTCP predictions and relevant dosimetric endpoints.^30^ It is equally important to communicate with patients about the potential benefits of IMPT, including reduced acute toxicity (e.g., mucositis, dysphagia) and lower risk of long‐term effects such as xerostomia or optic damage. Although access and insurance approval remain barriers in some systems, the growing body of dosimetric and clinical evidence supports IMPT use in appropriately selected cases.^31^ Clinicians should recognize that IMPT may not always outperform photons, particularly when targets encase critical structures, and hybrid strategies may be considered. As technology and availability improve, IMPT is expected to become a standard modality for skull base and head and neck tumors, offering effective tumor control with reduced collateral damage.

To optimize the use of proton therapy, future research should focus on refining selection criteria through model‐based approaches, incorporating normal tissue complication probability (NTCP) models and machine learning analysis of imaging and dosimetric data.^32^ This may help identify patient or tumor characteristics—such as specific skull base locations or favorable distances from OARs—‐that predict the greatest benefit from IMPT. Conversely, cases with extreme proximity between targets and OARs may be better suited to advanced photon techniques.^33^ Adaptive proton therapy strategies, including mid‐course replanning to account for anatomical changes, are also crucial to ensure that OAR sparing is maintained throughout the treatment course. Technological innovations such as intensity‐modulated proton arc therapy (IMPT‐arc) offer potential improvements in dose conformality for complex tumor geometries,^34^, ^35^ while FLASH proton therapy, if proven safe, could reduce toxicity beyond what conventional dosimetry predicts.^36^ Simultaneously, advances in MR‐guided adaptive photon radiotherapy are narrowing the dosimetric gap through daily re‐optimization.^37^ As both proton and photon modalities continue to evolve, ongoing comparative research will remain essential to guide evidence‐based decision‐making and tailor treatment to individual patient anatomy and clinical context.

Despite its strengths, this study has limitations. The small sample size (*n* = 12) limits statistical power, and some dose differences—such as for the optic pathways and the left parotids – may be clinically relevant but not statistically significant due to cohort size or anatomical variability. Additionally, the heterogeneity in tumor histology and location (e.g., chordoma, meningioma, nasopharyngeal carcinoma) may reduce generalizability, and subgroup analyses were not feasible. As a dosimetric study, we did not assess actual clinical outcomes, so dose reductions cannot be directly translated into improved tumor control or toxicity profiles without prospective validation. Moreover, this single‐institution analysis may reflect local planning practices, equipment, and expertise. Although consistent dose constraints were applied, differences in planning philosophy—robust CTV optimization for IMPT versus PTV‐based VMAT—introduce inherent bias, and planners were not blinded to modality. These factors highlight the need for caution in interpretation and support future multi‐institutional studies with clinical follow‐up.

## 5 CONCLUSION

In summary, the results demonstrate that proton therapy achieved significantly lower doses to critical OARs in the treatment of skull base tumors, without compromising target coverage. In particular, proton plans achieved significant reductions in the maximum doses to the brainstem (by more than 5 Gy on average, *p*<0.05) and spinal cord (by more than 10 Gy on average, *p*<0.05), as well as drastic dose reductions to anterior structures such as the eyes and lenses (by factors of 2–5, *p*<0.01). Optic nerves and chiasm doses were generally lower with protons, though the difference was case‐dependent and did not reach significance in aggregate due to cases of direct tumor involvement requiring high dose in both modalities. The volume of normal brain and head/neck tissues receiving low radiation doses was markedly smaller with protons, reflecting a sparing of integral dose. Our dosimetric findings strongly favor IMPT over VMAT for many skull base cases, but it remains for future research to confirm the extent of clinical benefit and to refine the application of particle therapy. By pursuing the above lines of inquiry, the radiation oncology community can ensure that the full potential of advanced technologies—be it protons, improved photon techniques, or even carbon ions—is realized for patients with challenging skull base tumors. The ultimate goal is to maximize tumor control while minimizing normal tissue harm, and ongoing research will pave the way toward more personalized and effective therapy for these patients.

## AUTHOR CONTRIBUTIONS

Yuanyuan Wang and Jiajun Zheng drafted the initial manuscript. Yuanyuan Wang, Hsiao‐Ming Lu, and Jin Gao contributed to the study conception and design. Lei Liu and Yuxiang Wang assisted with plan design and data analysis. Tengfei Long and Ming Liu participated in data analysis. All authors reviewed the manuscript and approved the final version.

## CONFLICT OF INTEREST STATEMENT

The authors declare that they have no competing interests.
